# Health status of seabirds and coastal birds found at the German North Sea coast

**DOI:** 10.1186/1751-0147-54-43

**Published:** 2012-07-19

**Authors:** Ursula Siebert, Philipp Schwemmer, Nils Guse, Timm Harder, Stefan Garthe, Ellen Prenger-Berninghoff, Peter Wohlsein

**Affiliations:** 1 Institut für Terrestrische und Aquatische Wildtierforschung, Stiftung Tierärztliche Hochschule Hannover, Werftstr. 6, Buesum, 25761, Germany; 2 Forschungs- und Technologiezentrum Westküste, Christian-Albrechts-Universität zu Kiel, Hafentoern 1, Buesum, 25761, Germany; 3 O.I.E., FAO und Nationales Referenzlabor für Aviäre Influenza, Institut für Virusdiagnostik, Friedrich-Löffler-Institut, Südufer 10, Greifswald-Insel Riems, 17493, Germany; 4 Institut für Hygiene und Infektionskrankheiten der Tiere, Justus-Liebig-Universität Giessen, Frankfurter Str. 85-89, Giessen, 35392, Germany; 5 Institut für Pathologie, Stiftung Tierärztliche Hochschule Hannover, Bünteweg 17, Hannover, 30559, Germany

**Keywords:** Seabirds, Coastal birds, Pathology, North Sea, German waters

## Abstract

**Background:**

Systematic pathological investigations to assess the health status of seabirds and coastal birds in Germany were performed. The investigation was conducted to obtain data on possible causes of decline in seabird and coastal bird populations.

**Methods:**

48 individuals of 11 different species of seabirds and coastal birds were collected by the stranding network along the entire German North Sea coast from 1997 to 2008, including mainly waders such as Eurasian oystercatchers (*Haematopus ostralegus*) and red knots (*Calidris canutus*) as well as seabirds such as northern fulmars (*Fulmaris glacialis*) and common scoters (*Melanitta nigra*).

For most birds (n = 31) found dead along the shore no obvious cause of death was evident, while 17 individuals were killed by collisions with lighthouses.

**Results:**

Overall, the nutritional status of the investigated birds was very poor, and the body mass in most cases was significantly lower compared to masses of living birds caught during the same periods of the year. This is partly linked to chronic parasitic or bacterial infections in different organs or to septicaemia. In some cases infections with zoonotic tuberculosis caused by *Mycobacterium* spp. were found. Avian influenza was not found in any of the collected birds.

**Conclusion:**

The presented data contribute to the evaluation of the health status of birds in the German North Sea. Moreover, they present an important tool for the assessment of potential pathogens with an impact on the health status of seabirds and coastal birds.

## Background

An accelerating and still ongoing decline in population sizes of several coastal bird species has been observed since the late 1990s [1]. Systematic pathological, microbiological and virological investigations on diseases and causes of death of seabirds and coastal birds are scarce for German waters. This information is necessary for the identification and quantification of effects exerted by various noxae and human activities.

Most published studies deal with specific infections such as parasitic load of selected organs in a chosen species [2] while necropsy studies are few, e.g. studies of acute haemorrhagic gastroenteropathy as a consequence of oiling and cachexia as primary cause of death [3,4]. Some investigations on avian influenza virus have been conducted in wild birds in German waters and an EU-wide systematic monitoring has been in place since 2004. Cases of the highly pathogenic avian influenza virus of subtype H5N1 (HPAIV H5N1), found in aquatic wild birds in 2006 and 2007, were scattered across Germany but concentrated in waters around Rügen, Mecklenburg-Western Pomerania, at Lake Constance, southwest Germany, and in small lakes in the central eastern parts of Germany [5].

Previous investigations conducted on other top predators such as harbour porpoises (*Phocoena phocoena*) and harbour seals (*Phoca vitulina*) from the same ecosystem as studied in the present investigation, the German North Sea, showed that animals suffered from parasitic and bacterial infections, particularly those of the respiratory tract [6-8]. Harbour porpoises from the Baltic Sea showed a significantly higher incidence of severe bacterial infections than those from less polluted waters around Greenland [9]. Polychlorinated biphenyl (PCB) concentrations in adult harbour porpoises were ten times lower in the Arctic than in the Baltic and North Seas [10,11]. These investigations showed that systematic health monitoring and toxicological studies can fundamentally contribute to the understanding of the causes of death, potential pathogens exerting pressure on populations and development of diseases in the wildlife population.

The present study gives results of pathological findings and their aetiology observed in different bird species found along the German North Sea coast.

## Material and methods

**Figure 1:**
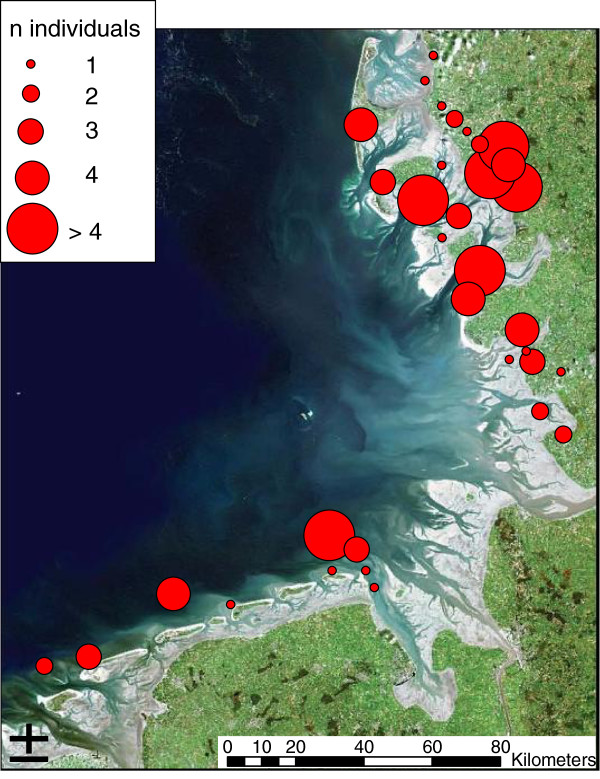
Location of birds found dead

The examined birds, either beached or found at the bottom of a lighthouse, were collected by different environmental organisations and state institutions of Schleswig-Holstein and Lower Saxony along the German North Sea coast (east of 7° and south of 55°) from 1997 to 2008 (Figure 1). A total of 48 individuals from 11 marine bird species were necropsied, comprising seven species of waders i.e. Eurasian oystercatcher (*Haematopus ostralegus,* n = 13), red knot (*Calidris canutus*, n = 9), curlew *(Numenius arquata*, n = 4) grey plover (*Pluvialis squatarola*, n = 2), dunlin (*Calidris alpina*, n = 1), bar-tailed godwit (*Limosa lapponica*, n = 2), ringed plover (*Charadrius hiaticula*, n = 1), three species of marine ducks, i.e. common scoter (*Melanitta nigra*, n = 7), northern fulmar (*Fulmarus glacialis*, n = 5). Carcasses of birds were stored at −20 °C until necropsy. The state of preservation was judged based on criteria described by [12] to assess the dehydration of the carcasses, and to estimate the courses of reduced body weight correctly.

### Gender and age distribution

Determination of age and gender was conducted based on the criteria described by [13] and [14].

### Nutritional status

Nutritional status was assessed based on the amount of subcutaneous and visceral fat and atrophy of sternal muscle according to [12], resulting in four categories of nutritional stage: 0 = cachexia, 1 = poor, 2 = moderate, and 3 = good. In addition, sufficiently dry individuals (all northern fulmars were too wet) were weighed and results were compared to values of birds captured along the German Wadden Sea coast for ringing purposes (Hälterlein personal communication). Only individuals classified as “very fresh” (n = 7), “fresh” (n = 22) and “rather fresh” (n = 5) according to the classification given by [12] were weighed to avoid potential underestimation of body weights due to decomposition and dehydration. For common scoter and shelduck, values were taken from the literature [15].

### Post mortem investigation

The carcasses were examined for external lesions and external presence of oil. The percentage of body surface covered by oil was judged as described by [12]. All organ systems were examined macroscopically [16].

### Histopathological investigations

Samples for histopathology were collected from lung, trachea, stomach, intestine, oesophagus, liver, adrenal gland, kidney, testis, ovary, spleen, thymus, lymph nodes, heart, aorta, skeletal muscle, skin, feathers, brain, spinal cord and from grossly changed organs and tissues. The samples were fixed in 10% neutral buffered formalin and embedded in paraffin wax. Sections of 5 μm were stained with haematoxylin and eosin (HE). Selected sections were stained additionally with Periodic acid-Schiff (PAS) reaction, Ziehl-Neelsen´s or Kongo red stain.

### Microbiological and virological investigations

Lung, liver, kidney, spleen, intestine, lymph nodes and tissues with macroscopical lesions were examined microbiologically as described by [8]. Possible *Mycobacterium* spp. were identified by Dr. Tobias Eisenberg at the State Laboratory of Hessen, Germany using polymerase chain reaction (PCR) according to [17] and [18]. Tracheal and intestinal swabs were submitted for virological investigations on avian influenza to the O.I.E., FAO and National Reference Laboratory for Avian Influenza, Friedrich-Loeffler Institute, Germany. RNA was extracted from swabs and analysed for avian influenza virus-specific RNA by real-time (RT) PCR, as previously described [19]. In addition, samples for toxicology (muscle, liver, kidney, fat) and content of stomach were preserved.

### Statistics

Generalised Linear Models (GLM; [20]) were used to test for differences in body condition of all species (as well as for single species if sample sizes were sufficiently large) between age and gender as well as between victims of light house collisions and those with other causes of death. Due to the left-skewed distribution caused by many zero values a quasi-Poisson distribution was taken as a basis. The masses measured were compared with those of living individuals captured for ringing purposes (= expected weights; (Hälterlein personal communication). To account for seasonal differences in body mass, we only compared masses of living and dead birds caught or collected during the same seasons. The comparison was performed by means of a χ²-test for goodness of fit [21]. All tests were conducted with the freely available statistics software R, version 2.8.1 [22].

## Results

### Gender and age distribution

The gender and age distributions of the examined birds are shown in Table 1. Thirtheen birds were mature females and 25 mature males, 6 were immature females and 2 immature males. The sex and gender of one Curlew were not identified.

**Table 1:** Gender and age structure of bird species from the German North Sea (numbers in parentheses indicate the number of birds found after collision with a lighthouse)

**Name**	**mature**		**immature**		**age/gender unknown**	**Sum**
	**female**	**male**	**female**	**male**		
Oystercatcher	2	8	3	-	-	13 (1)
Red knot	5	4	-	-	-	9 (7)
Common scoter	-	4	2	1	-	7 (0)
Northern fulmar	1	2	1	1	-	5 (0)
Curlew	-	3	-	-	1	4 (3)
Shelduck	3	-	-	-	-	3 (0)
Grey plover	-	2	-	-	-	2 (1)
Bar-tailed godwit	2	-	-	-	-	2 (2)
Dunlin	-	1	-	-	-	1 (1)
Common eider	-	-	-	1	-	1 (0)
Ringed plover	-	1	-	-	-	1 (0)
	**13**	**25**	**6**	**2**	**1**	**48**

### Nutritional condition

The majority of investigated animals were in a reduced nutritional condition (Table 2). Particularly the northern fulmar and common scoter showed cachexia. The best nutritional status was found in red knots without any emaciated individuals. When considering all bird species, animals killed by light house collision showed a significantly better nutritional condition compared to stranded individuals (GLM: t = 2.4; *P* = 0.023; R² = 11.1). Differences in nutritional status in red knots killed due to collision and those that were stranded were not significant (t = 0.3; *P* = 0.7). Sample sizes for separate tests of other bird species were too low.

**Table 2:** Comparison of the mean measured (minimum and maximum ranges in brackets) and expected body masses of birds investigated

**Species**	**Mean measured mass [g]**	**Mean expected mass [g]**	**X²**	**df**	** *P* **
Oystercatcher	396.6 (288.7 – 586)	527.1	455.3	9	< 0.001
Red knot	141.8 (126–150)	146	7	4	n.s.
Common scoter	727 (655–851)	1057.1	777.9	6	< 0.001
Curlew	578.3 (404–890)	751	288.3	1	< 0.001
Grey Plover	210.1 (184.7–235.4)	251.1	22.5	1	< 0.001
Bar-tailed godwit	232.2 (186.6-277.5)	418.5	175.8	1	< 0.001

n.s.: not significant, df = degrees of freedom.

When considering all bird species, there was no significant difference in body condition between adults and immatures (t = 0.5; *P* = 0.1). The same holds true for oystercatchers (t = 0.5; *P* = 0.3) as well as for common scoters (t = 0.7; *P* = 0.6). Sample sizes for other species were too low.

To test for gender-related differences we first performed a test using all birds over all species investigated. There was no significant difference in body condition between genders (t = 1.1; *P* = 0.3) when including all individuals. The same holds true separately for oystercatchers (t = 0.9; *P* = 0.4), northern fulmars (t = 1.5; *P* = 0.3), red knots (t = 0.6; *P* = 0.6) and common scoters (t = 0.3; *P* = 0.8).

Body masses of investigated individuals were significantly lower than in individuals caught for ringing or as stated in literature in oystercatchers, common scoters, curlews, grey plovers and bar-tailed godwits, whereas differences in red knots were insignificant (Table 2). For the other species no tests could be conducted.

### Contamination with oil

Three of 48 individuals investigated (6.3%) showed external contamination with oil. One of the five northern fulmars showed a small patch of oil (covering less than 1% of its plumage). The single common eider as well as one of the five common scoters revealed a contamination of 5% of their plumage.

### Pathological findings

#### Oystercatcher

**Figure 2:**
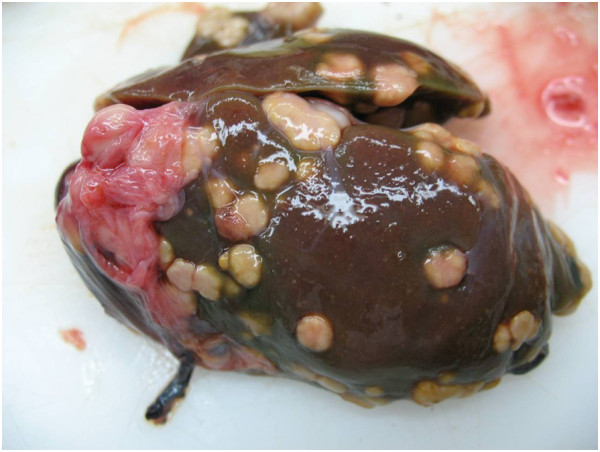
Severe granulomatous hepatitis in an oystercatcher (size of liver = 8.2 cm)

**Figure 3:**
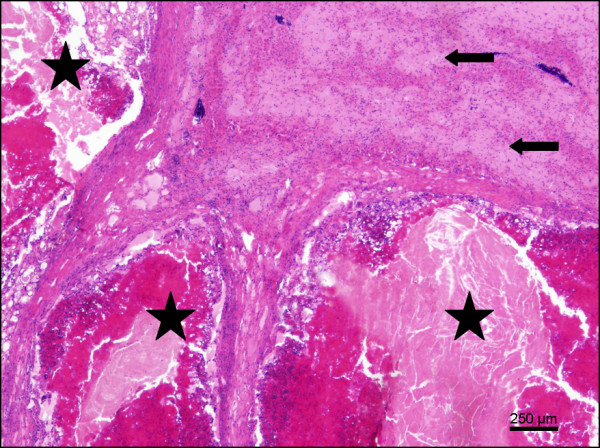
Severe, chronic granulomatous hepatitis in an oystercatcher (asterisks); adjacent hepatic tissue with atrophy due to significant deposition of amyloid (arrows); HE; bar = 250 μm

In one out of 13 oystercatchers a severe diffuse granulomatous necrotising pneumonia was found also suffering from granulomatous necrotising splenitis and hepatitis (Figure 2). The granulomatous and catarrhal inflammation of the intestinal tract in this individual bird was histologically associated with fragments of parasites, which could not be further identified. Five oystercatchers displayed severe granulomatous and suppurative necrotising hepatitis (Figure 3). In one individual a granulomatous serositis of the intestine was found and in another animal amyloidosis. The cause of these findings was bacterial infection in three cases (see Table 3). Two oystercatchers showed histological evidence of coccidia in the kidneys and mild non-suppurative nephritis as well as hyaline droplet storage nephrosis.

**Table 3:** Microbiological findings in birds from the German North Sea

	**Red knot**	**Oyster catcher**	**Flumar**	**Common scoter**	**Other**
*Clostridium perfringens*	A5	A3	A2	A5	A1
*Escherichia coli*	A2	A3, B1, D1, F2, G1	A1	A1, C1, E1, G1	D1, G1
Beta-haemolytic streptococci			A1, E1, F1, G1		
*Mycobacterium avium*		D1		A2, D2, E2, F2, G2	A1, D1, E1, F1, G1

1-5 number of positive animals, A intestine, B cloacae, C bones, D liver, E lung, F spleen, G kidney.

Fractures and articular dislocation with surrounding haemorrhage indicating death from trauma were found. Additional chronic lesions of the muscles (e.g. calcification, fibrosis) indicated traumatic events earlier in life. In two cases death resulted most likely from light house collision.

Lesions of the skin and subcutis were rare. Mites between the quills, dermatitis, panniculitis and folliculitis were diagnosed in one case each. A papilloma was found on the leg of one animal. Further characterisation was impossible due to poor preservation of the carcass.

#### Northern fulmar

Only few lesions were found in the 5 investigated fulmars. Histologically, nematodes were found in the oesophagus, the true and muscular stomach. In two cases, granulomatous and catarrhal gastritis was found. One animal displayed focal non-suppurative nephritis.

#### Red knot

Pathological findings in the respiratory tract of 9 red knots displayed unidentifiable foreign material in the deeper respiratory tract of one individual. In two cases, histological investigations revealed nematodes in the small intestine. Granulomatous to necrotising serositis associated with acid-fast bacilli was found in one cachectic animal. Fractures or dislocated joints with haemorrhage in the musculature and subcutis indicated acute trauma in four birds, all of which had been classified as collision victims.

#### Common scoter

Seven common scoters displayed severe pneumonia, gastroenteritis, hepatitis, splenitis, nephritis and/or encephalitis. The character of the inflammation varied between granulomatous, ulcerative, necrotising, suppurative and non-suppurative. Some animals showed inflammation in several organs, indicating a septicaemic infection. The severe chronic organ lesions were associated with a reduced nutritional status of the common scoters. Nematodes were histologically diagnosed in one case in the stomach, and trematode eggs were observed in the intestine of three cases.

#### Other bird species

A smaller number of ringed plovers, grey plovers, bar-tailed godwits, dunlins, common eiders, shelducks and curlews were investigated.

**Figure 4:**
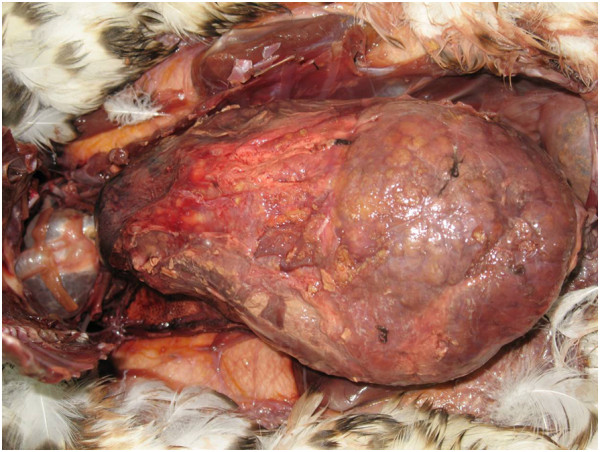
Pyogranulomatous inflammation of all internal organs and serositis in a shelduck (size = 11 cm)

In addition to signs of trauma, Kentish plover, grey plover, bar-tailed godwit and dunlin showed only mild lesions such as catarrhal gastritis or proliferation of the bile ducts. In contrast, common eider, common shelduck and curlew showed more frequent and severe lesions. Severe pyogranulomatous, fibrinous and necrotising serositis, perihepatitis, hepatitis, splenitis and myocarditis (Figure 4) resulted in adhesions in all organs. Histologically, nematodes were observed in the stomach, cestodes and trematodes were found in the intestine as well as arthropods in the skin.

### Microbiological findings

**Figure 5:**
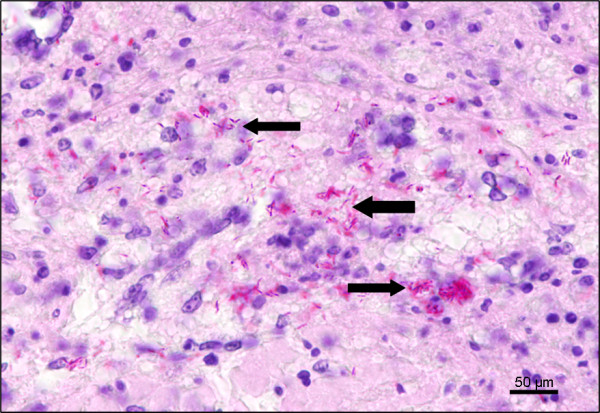
Lung of a common scoter; numerous acid fast bacilli within the inflammatory exudate; Ziehl-Neelsen´s stain; bar = 50 μm

Microbiological investigations revealed a total of 31 different bacteria and fungi. In 99 organs no growth of bacteria or fungi was detected. Beside bacteria of likely insignificance *(*e.g. *Aeromonas* sp*. Branhamella* sp.*, Pseudomonas* sp*.* and gamma-streptococci*)* four species of potentially pathogenic bacteria were isolated, including *Clostridium perfringens, Escherichia coli,* streptococci and acid-fast bacilli (Table 3). In addition, *Aspergillus niger* was found. Bacteria and fungi isolated were found in direct association with pneumonia, gastroenteritis, hepatitis, nephritis, splenitis, serositis and septicaemia. Acid-fast bacilli (Figure 5) were found in oystercatchers, common scoters, shelducks, common eiders and bar-tailed godwits in several organs in culture but also histologically in granulomatous lesions stained with Ziehl-Neelsen s method (8 of 48 animals, i.e. 17%). These bacteria were identified as *Mycobacterium avium* subsp*. avium*.

### Virological findings

RNA specific to avian influenza virus was not detected in any of the investigated birds. Use of an internal control ruled out false-negative reactions. No indication of viral infections was found in necropsy and histological investigations.

### Main causes of disease and death

Lesions in oystercatchers comprised cachexia (6/13), inflammation of several internal organs (2/13), hepatitis (2/13), in one case associated with amyloidosis, serositis (1/13) and trauma (1/13). In Northern fulmars cachexia was the main morphological change (4/5). Besides serositis (1/9) and cachexia (1/9) traumatic changes were the main lesions (6/9) in red knots. In common scoters morphological changes consisted of cachexia (3/7), septicaemia (2/7), inflammation of several internal organs (1/7) and nephritis (1/7). In other species lesions comprised cachexia (3/14), inflammation of several internal organs (2/14), septicaemia (1/14), enteritis (1/14), serositis (1/14), and trauma (1/14).

In summary, 17 birds (35%) most likely died solely from cachexia, because organ or tissue lesions were not detectable. In 15 birds (31%) inflammation of one or several organs was found, mostly associated with cachexia. In one oystercatcher granulomatous inflammation was associated with amyloid depositions in several organs. These findings are regarded as possible contributing factors leading to death. In 8 individuals (17%) with obviously good body conditions death resulted from trauma. The death of 8 other birds (17%) was due to circulatory failure of unknown cause.

## Discussion

A systematic pathological investigation of both, coastal and seabirds from German waters, has not yet been performed. A decline in several avian species in the Wadden Sea has been observed, prompting the initiation of this pilot study on stored bird carcasses.

One of the most important findings of these investigations was multi-organ infection with *M. avium* subsp. *avium*. In the Netherlands, Smit et al. [23] examined 11,664 individuals from 20 different bird species (predominantly non-passerines) for *Mycobacterium* infection. The bacterium was found in 82 of these individuals (0.7%). Eurasian Buzzard was the species most frequently affected. Oystercatchers and shelducks were the only two species in the study of Smit et al. [23] which were also investigated in our study. One out of 95 oystercatchers (1.1%) and two out of 64 shelducks (3.1%) examined by Smit et al. [23] were infected with *Mycobacterium* sp*.* In comparison, 17% of the individuals in our study revealed an infection with *Mycobacterium* sp. Knowledge about the distribution and population relevance of tuberculosis in seabirds and coastal birds is currently unavailable. Comprehensive studies, including molecular, biological and serological tests, are urgently needed. Compared to *M. bovis* or *M. tuberculosis, M. avium* is a zoonotic bacterium with lesser virulence for humans. Usually it causes localised lesions in humans and animals, e. g. restricted to the lung or lymph nodes. Persons handling carcasses and live animals are urged to take appropriate precautions against infection.

Of all countries bordering the North Sea, Belgium has produced by far the largest number of studies of comparable quality regarding pathology of seabirds and coastal birds. Jauniaux et al. [4] conducted histopathological, bacteriological, parasitological and toxicological analyses of seabirds, particularly guillemots (*Uria aalge*) found dead on beaches from 1992 to 1995. Cachexia, acute haemorrhage of the gastrointestinal tract and oil contamination were the most frequent causes of death. The authors correlated the results with age, sex and origin (pelagic *vs.* coastal habitat) of the birds. Particularly seabirds living in the pelagic zone were in an advanced state of cachexia mostly, however, in connection with oil contamination. Our data, in contrast, indicate an equally poor nutritional status in coastal and seabirds. Very similar results were obtained by Jauniaux et al. [3], who investigated a total of 133 individuals (guillemot, oystercatcher, kittiwake, razorbill and herring gull) found dead along the Belgian coast.

Of 67 guillemots found dead along the Belgian North Sea shore from 1993 to 1994, 70% showed signs of cachexia in addition to signs of haemorrhagic gastrointestinal disease connected to oil contamination [24]. It is important to note, however, that our study also included a higher percentage of cachectic birds without lesions. Toxicological studies of the birds investigated in Belgium did not reveal increased concentrations of pollutants, which may have illuminated the findings [24].

In comparison to the studies from Belgian waters mentioned above, which showed acute haemorrhagic gastrointestinal disease to be the most frequent lesion, the birds we examined revealed a much wider range of lesions. The proportion of oil-contaminated individuals was in any case considerably lower in our investigation than in Belgian studies [24] possibly influenced by the different bird species composition in which oil contamination may have caused most of the lesions.

Parasites played a minor role in the material investigated. Several other studies were focused only on the occurrence of parasites (e.g. Borgsteede [2]). A parasitological investigation of the gastrointestinal tract in 25 common eiders collected in the Netherlands from 1976 to 1991 [25] showed that they were infected with 5 nematode, 12 trematode, 1 cestode and 1 acanthocephale species. In some animals, up to 100,000 parasites were found. The authors postulated that this may have had a negative effect on the nutritional state. The differences between other investigations and the present study may result from regional or temporal variations. On the basis of the findings presented here, high parasitic infestation may be ruled out as a cause of reduced nutritional status and decreased population size in the birds investigated. A larger sample size from a longer time period should be investigated to confirm this conclusion.

The importance of cachexia is illustrated by the eider mass die-off during the winter of 1999/2000 when approximately 21,000 birds died due to starvation in the Netherlands alone [26]. While the study mentioned comprised only a parasitological investigation, it was rather clear that the mass die-off was caused by the poor body condition of the birds. In this connection, the high percentage of cachectic individuals in our study should be considered an important indicator of a potentially poor nutritional status in many species. One third of the birds of the present study died from infectious diseases, raising the question about potential immunosuppression or high infection pressure. As the infections were accompanied by cachexia in all birds the ultimate cause of death might have been a combination of lesions and starvation; although it is not clear whether initial starvation led to increased vulnerability to infections or vice versa. The only species that showed a comparatively good nutritional status was the red knot. Among all species, most individual red knots were collected after collision with a lighthouse. This may explain the comparably good nutritional and health status compared to the other bird species. However, several of the individuals killed by collision (both in red knot as well as in bar-tailed godwit) showed severe lesions of inner organs as well. It can be assumed that birds already diseased might be more likely to collide with buildings as weakened birds might show reduced flight ability and are probably more likely to collide with structures in strong winds. However, there is no evidence for this assumption. With respect to the comparison of measured and expected body weights it needs to be considered that decomposition of carcasses may have led to underestimation of body weights of the stranded birds. However, we tried to account for this potential bias by exclusively weighing fresh carcasses and rejecting measurements of older carcasses. In the context of some Belgian studies, an attempt was made to demonstrate the connection between cachexia and heavy metals and/or PCB burden in several hundred guillemots collected on beaches over the course of six years: Cachectic individuals showed a higher burden of contaminants in kidney and liver, also of breast muscle in some birds, than individuals in a better nutritional state [27,28]. The higher pollutant burden is considered to be at least an additional stressor facilitating cachexia [27] and/or indicates a transfer or mobilisation of such pollutants during starvation [28]. Pollutant analyses for the birds presented here are unfortunately not yet available.

Indications of avian influenza or other viral infections were not found in the specimens available for our investigation. Highly pathogenic avian influenza virus of subtype H5N1 (HPAIV H5N1) in wild birds was found in 2006 and 2007 but not in birds from the Wadden Sea of Germany [5].

The influence of seasonal changes on the condition of various organ systems is firmly established [29-34]. The variability is further increased by other environmental factors capable of impacting body condition and health status: Weather [26,32] and quality of the feeding habitat [35] should be mentioned here. This high variability may only be reduced through access to a larger sample size. In addition this would also allow analysing data further in relation of the migration status of the birds.

## Conclusions

The findings presented here show that pathological investigations are important for an understanding of the health status in coastal birds as well as seabirds. However, it is important to combine those with specific tests, e.g. on the immune system which need to be conducted in alive individuals. Sample sizes of the current study should be increased urgently because the present material originated from a wide range of ecologically different bird species from different regions and seasons, as well as migratory phases and moulting stages, and was collected over several years, thus resulting in a high variability of the data.

## Competing interests

The authors declare that they have no competing interests. No animal experiments were conducted.

## Authors’ contributions

US, PS and NG conducted the postmortem examinations on the birds. PW performed the histological, EPB the microbiological and TH the virological investigations. US and PS drafted the manuscript and the others authors contributed to the manuscript. All authors read and approved the final manuscript.
